# Safety and feasibility assessment of implantable chest wall venous access port for patients with acute leukemia

**DOI:** 10.3389/fonc.2026.1749957

**Published:** 2026-01-28

**Authors:** Peng Ke, Min Liao, Fan He, Ying Xu, Guoqiang Li, Lina Hu, Chun Feng, Ruiting Zhang, Yanhong Ding, Jihao Zhou, Jialing Lu, Yixuan Cao

**Affiliations:** 1 Department of Hematology, Shenzhen People’s Hospital, The First Affiliated Hospital, Southern University of Science and Technology, The Second Clinical Medical College, Jinan University, Shenzhen, Guangdong, China; 2 Department of Invasive Technology, Shenzhen People’s Hospital, The First Affiliated Hospital, Southern University of Science and Technology, The Second Clinical Medical College, Jinan University, Shenzhen, Guangdong, China; 3 Shanghai Municipal Hospital of Traditional Chinese Medicine, Shanghai University of Traditional Chinese Medicine, Shanghai, China

**Keywords:** acute leukemia, feasibility, safety, TIVAP-related complications, totally implantable venous access ports

## Abstract

**Background:**

Totally implantable venous access ports (TIVAPs) are widely used in chemotherapy for solid malignant tumors. However, there are currently few data on the application of TIVAPs in patients with acute leukemia. The purpose of this study was to evaluate the safety and feasibility of TIVAPs in patients with acute leukemia.

**Methods:**

A single-institute retrospective review was performed on 94 adult patients with acute leukemia who underwent TIVAPs at Shenzhen People’s Hospital between January 2020 and April 2025. Baseline characteristics and TIVAP-related complications were evaluated.

**Results:**

Among of 94 patients, all TIVAPs were successfully implanted. No intraoperative complications occurred. Early postoperative complications (less than 30 days postoperatively) occurred in five (5.3%) patients, including local subcutaneous hematoma (n=3, 3.2%) and infection (n=2, 2.1%). Only two patients (n=2, 2.1%) developed late postoperative complications (more than 30 days postoperatively), including one catheter-related deep vein thrombosis and one catheter dysfunction. Three of 94 patients (3.2%) required premature TIVAPs removal due to localized bleeding complications or catheter-related bloodstream infection. As of June 1, 2025, the median dwelling time of TIVAPs was 26.6 months (range: 0.1-76.4 months). The 2-year of cumulative hematoma-free catheter survival rates was 96.8% ± 1.81%. The 2-year of cumulative adverse event-free catheter survival rates was 93.5% ± 2.55%.

**Conclusions:**

Our study shows that TIVAP can provide a safe and effective system for long-term intravenous treatment of patients with acute leukemia. Therefore, TIVAP is another potentially effective and safe option for patients with acute leukemia.

## Introduction

The incidence of malignant diseases is on the rise worldwide, increasing from 18 million in the year 2018 to 19.96 million in the year 2022 (1, 2). Intravenous chemotherapy is one of the important treatment strategies for the vast majority of cancer patients. However, due to the particularity of chemotherapy drugs, many intravenous chemotherapies require a central venous device (CVD). Besides administering anticancer therapy, CVDs are used to administer blood, hydration, blood products and parenteral nutrition (3). Reliable and safe access to a central vein over a long period is of great important in the process of tumor treatment. Peripherally inserted central catheters (PICCs) and totally implantable venous access ports (TIVAPs) are the most common venous access options for intravenous chemotherapy.

PICC is a long vascular device that is inserted into a peripheral vein of the arm and the catheter tip is then inserted into the superior vena cava. While TIVAP is a catheter attached to a reservoir (a port chamber placed in a subcutaneous pocket) that is commonly inserted into the subclavian vein or the internal jugular vein and the catheter tip is then also inserted into the lower 1/3 of the superior vena cava (4). PICCs have historically seen increases in use, especially among patients with malignant hematological diseases, due to the ease of insertion by nursing teams outside the operating theatre, versatility, alleged greater safety and cost effectiveness (5). However, PICCs are associated with higher risk for catheter-related deep venous thrombosis and other adverse events (infection, occlusive and mechanical problems) when compared with TIVAPs (6–8).

In recent decades, the use of TIVAP as an alternative to traditional PICC has increased, especially in non-hematological tumors. Many cancer patients prefer TIVAP over PICC, because TIVAP is more convenient, comfortable and has less negative impact on the quality of life (9). Intravenous ports have been shown to have an advantage over PICC in terms of health economics for a 6 and 12-month dwell time because of their lower maintenance costs and fewer complications (10). Leukemia is a common hematologic malignancy for which intravenous chemotherapy is a cornerstone of treatment. This is particularly true for acute leukemia, a disease that requires multiple cycles of intensive chemotherapy (e.g., cytarabine and daunorubicin), frequent transfusions, intravenous nutritional support, and often entails treatment durations exceeding three months. Consequently, establishing long-term, reliable, and safe central venous access is of paramount importance in the management of acute leukemia. However, in clinical practice, due to the particularity of patients with acute leukemia (abnormal white blood cells, low platelets and abnormal coagulation function), hematologists still tend to choose PICC as the venous access to reduce the risk of catheterization.

At present, there is on the application of TIVAPs in patients with acute leukemia. The purpose of this study was to evaluate the safety and feasibility of TIVAPs in patients with acute leukemia. Our results might help further determine that this technique can serve as another potentially effective option for acute leukemia patients.

## Patients and methods

A total of 94 adult patients with acute leukemia received TIVAPs at Shenzhen People’s Hospital between January 2020 and April 2025, primarily for the administration of chemotherapy, including induction, consolidation, maintenance and palliative chemotherapy. All patients provided written informed consent for the protocol, which was approved by hospital’s Ethics Committee of the Shenzhen People’s Hospital.

### Protocol for TIVAP surgical procedure

Only one model of TIVAP device (Bard, 8806061, 6Fr; Bard Access Systems, Inc., Utah, USA) was used in this study. All procedures were performed by interventional physicians in a sterile operating room under local anesthesia. Using the Seldinger technique under real-time ultrasound guidance, the subclavian vein puncture was performed. The catheter was typically introduced via the right subclavian vein access site. A separate infraclavicular incision (approximately 2 cm in length, parallel to and 1–2 fingerbreadths below the clavicle) was made to create a subcutaneous pocket that precisely accommodated the port reservoir. The catheter was then tunneled subcutaneously from the venous access site to the pocket, connected to the reservoir, and the assembly was placed into the pocket after verifying patency and absence of leakage by flushing. Catheter tip was advanced to the cavoatrial junction, with final position confirmed by intraoperative fluoroscopy. The incision was closed in layers using 4–0 absorbable suture and covered with a sterile dressing. According to institutional protocol, only experienced nursing staff accessed the port. After each use and at regular maintenance intervals, the port was flushed with normal saline and locked with heparinized saline. For long-term unused ports, patency was verified every 3 months. According to institutional protocol, only experienced nursing staff accessed the port. After each use and at regular maintenance intervals, the port was flushed with normal saline and locked with heparinized saline. For long-term unused ports, patency was verified every 3 months.

### Data collection

Research data were collected retrospectively using a structured, standardized electronic case report form (e-CRF) specifically designed for this study. Data were extracted from our hospital’s prospectively maintained electronic medical records (EMR) system, which integrates clinical, laboratory, procedural, and nursing documentation. Based on the time of occurrence, postoperative complications were divided into immediate (within 24 hours), early (≤30 days) and late complications (>30 days). Based on previous studies on complications after TIVAP implantation, the complications were broadly classified as: wound complications (hemorrhage, wound dehiscence, delayed incision healing and so on), mechanical complications (catheter dysfunction, catheter malposition and so on), infectious complications (local infection, catheter-related bloodstream infection and so on), blood vessel complications (artery trauma, vessel thrombosis and so on) and others (pneumothorax, unplanned extubation and so on). We conducted follow-up patient satisfaction surveys after surgery, focusing on intraoperative pain levels, postoperative comfort, and the impact on daily life.

### Definitions

Cutaneous site infection was defined as redness and swelling, skin temperature arisen, tenderness and exudate around the port. Catheter-associated infections were defined according to Infectious Diseases Society of America criteria (11). Catheter obstruction was defined as the inability to be aspirated or flushed through the catheter. Catheter obstruction was included in the analysis only when urokinase was needed to resolve the occlusion. Time to complication (TTC) was defined as the period from TIVAP implantation to the day of onset of complication. Pain was graded according to the Numeric Rating Scale (NRS).

### Statistical analysis

All variables were tested with the Shapiro-Wilk test for normality, and verified for completeness. Descriptive statistics were presented in various forms, including mean ± standard deviation (range), median (range), and frequency (%). All analyses were performed with statistical software SPSS 25.0 windows software (SPSS, Chicago, IL). The deadline for follow-up was June 1, 2025, with a median follow-up time of 26.6 (0.1-76.4) months.

## Results

### Patient characteristics

Among the 94 patients, all patients underwent only one central venous port implantation procedure, with 59 males and 35 females. The age of 94 patients ranged from 18 years to 77 years, with a median of 43 at the time of surgery. All patients suffered from leukemia, including 67 acute myeloid leukemia (AML) and 27 acute lymphocytic leukemia (ALL), and intravenous chemotherapy was the indication for TIVAP implantation. Seventy-four (78.7%) cases were newly-diagnosed acute leukemia patients with no history of chemotherapy, while the remaining patients had already received oral and/or intravenous chemotherapy before the implantation of TIVAPs. The platelet count was elevated from the median of 71 × 10^9^/L (range: 3-406 × 10^9^/L) on admission to that of 76 × 10^9^/L (range: 10-554 × 10^9^/L) before surgery. There were 23 patients (24.5%) with platelets less than 50 × 10^9^/L and 71 patients (75.5%) with platelets higher than 50 × 10^9^/L at the time of surgery. The median neutrophil count was 2.12 (range: 0.10-67.50). Among them, 66 patients (70.2%) had mild neutropenia (absolute neutrophil count, ANC: 1000–1500 cells/μL), 13 patients (13.8%) had moderate neutropenia (ANC: 500–999 cells/μL), and 15 patients (16.0%) had severe neutropenia (ANC: <500 cells/μL). In addition, low fibrinogen levels (normal range: 2–4 g/L) were observed in 9 patients (9.6%), with a median of 1.6 g/L (range: 1.2-1.97 g/L). A prolonged activated partial thromboplastin time (APTT; normal range: 28–43 seconds) was observed in 12 patients (12.8%), with a median of 49.35 seconds (range: 44.1-58.7 seconds). In no case did the APTT exceed 1.5 times the upper limit of normal. An elevated international normalized ratio (INR; normal range: 0.8-1.3) was observed in 9 patients (9.6%), with a median of 1.43 (range: 1.35-1.50). Among them, thirty-five patients with thrombocytopenia received blood transfusions before surgery in order to ensure smooth operation. The general characteristics of the study group are summarized in the **Table 1**.

**Table 1:** Patient demographic and clinical characteristics.

Characteristic	Whole cohort (N = 94)
Age, years, median (range)	43 (18-77)
Gender, n (%)	
Male	59 (62.8)
Female	35 (37.2)
Disease type, n (%)	
AML	67 (71.3)
ALL	27 (28.7)
Treatment phase, n (%)	
Induction	74 (78.7)
Consolidation/maintenance/salvation	20 (21.3)
WBC count, ×10^9^/L, median (range)	6.12 (0.85-169.77)
Neutrophils count, ×10^9^/L, median (range)	2.12 (0.10-67.50)
Neutropenia, n (%)	
Mild	66 (70.2)
Moderate	13 (13.8)
Severe	15 (16.0)
PLT count before surgery, n (%)	
< 50×10^9^/L	23 (24.5)
≥ 50×10^9^/L	71 (75.5)
APTT, n (%)	
< 43 s	82 (87.2)
≥ 43 s	12 (12.8)
International Normalized Ratio	
< 1.3	85 (90.4)
≥ 1.3	9 (9.6)
Fibrinogen level, n (%)	
< 2 g/L	9 (9.6)
≥ 2 g/L	85 (90.4)
Preoperative antibiotics, n (%)	
Yes	31 (33.0)
No	63 (67.0)
Previous solid tumors, n (%)	
Yes	13 (13.8)
No	81 (86.2)
Deep venous thrombosis/cardiovascular history, n (%)	
Yes	6 (6.4)
No	88 (93.6)
Multiple CVAD implantation, n (%)	
Yes	19 (20.2)
No	75 (79.8)
Follow-up of TIVAP, months, median (range)	26.6 (0.1-76.4)

AML, Acute myeloid leukemia; ALL, Acute lymphocytic leukemia; WBC, white blood cell counts; PLT, platelet; CVAD, central venous access device; APTT, activated partial thromboplastin time.

### Surgical treatment

In all of the patients, venous access was achieved using the Seldinger method and the catheter was inserted through the right subclavian vein with a success rate of 100%. Vascular puncture was successful in the first attempt for 89 patients (94.7%), and in the second attempt for 5 patients (5.3%). The average time of operation was 28 ± 3 minutes (range: 23–48 minutes). No procedural related complications occurred, including pneumothorax, inadvertent artery puncture, air embolism, arrhythmia, nerve damage, and persistent bleeding at the puncture site. All patients started using TIVAPs two hours after operations.

### Follow-up outcomes

As of June 1, 2025, the median dwelling time of TIVAPs was 26.6 months (range: 0.1-76.4 months), with a total of 82430 catheter-days. TIVAP-related complications were identified in 7 patients (7.4%) in this study, corresponding to an incidence of 0.085 complications/1000 catheter-days. All patients experienced no intraoperative complications and passed the perioperative period safely within 24 hours. Early postoperative complications (less than 30 days postoperatively) occurred in five (5.3%) patients, including local subcutaneous hematoma (n=3, 3.2%) and infection (n=2, 2.1%), with no occurrence of port flipping and wound dehiscence. Three cases of local subcutaneous hematoma were located in the pocket of the TIVAPs and occurred within 7 days after surgery. Two of the patients were managed with local compression hemostasis and dressing changes, eventually resolving spontaneously. The third patient’s TIVAP was removed due to failed hemostasis. Two patients (2.1%) developed infections after placement of the infusion port: one case of catheter-related bloodstream infection (CRBSI) and one of local pocket infection combined with CRBSI. The patient with CRBSI experienced recurrent fever and chills 8 days post-TIVAP implantation. Blood cultures from the TIVAP and peripheral vein both grew *Escherichia coli*, with TIVAP blood cultures becoming positive 2 hours earlier than peripheral blood cultures. Given severe infection and underlying leukemia-related immunosuppression, the TIVAP was immediately removed and broad-spectrum antibiotics initiated. Subsequently, the patient’s infection was effectively controlled, and blood cultures from peripheral venous were negative twice. Another patient suffered from pocket infection 28 days post-TIVAP implantation, presenting with local redness, swelling, warmth, along with systemic symptoms such as chills and shivering. Cultures from venous blood, infusion port blood, and pocket secretions all grew *Staphylococcus aureus*. The patient immediately removed the infusion port and improved one week after empirical anti-infection treatment. The TIVAP was promptly removed, with intensification of local wound care and systemic intravenous anti-infective therapy. Clinical symptoms improved within one week.

During the follow-up period, only two patients (n=2, 2.1%) developed late postoperative complications (more than 30 days postoperatively). One patient developed catheter-related deep vein thrombosis (CR-DVT) 82 days after TIVAP implantation. The patient was diagnosed via contrast-enhanced CT and received anticoagulation with sequential low-molecular-weight heparin followed by rivaroxaban until thrombus resolution. Another patient developed intraluminal catheter obstruction on postoperative day 114, manifesting as failed blood aspiration and fluid infusion. The patient exhibited no limb swelling or pain, and vascular ultrasound showed no thrombosis; catheter dysfunction was confirmed as the final diagnosis. Catheter patency was restored via thrombolysis with urokinase (5000 IU/ml) and a positive pressure lock for 30–60 minutes. No other complications such as catheter malposition or fracture, catheter blockage, pinch-off syndrome and drug extravasation were observed.

In addition, 35 patients developed bloodstream infections while receiving chemotherapy post-TIVAP implantation. Nine infections occurred within 30 days post-implantation, and twenty-six occurred beyond 30 days post-implantation. All bloodstream infections occurred more than 48 hours after TIVAP implantation. In addition, all infections occurred during the period of myelosuppression, with no local signs of infection (e.g., tenderness, erythema, swelling, or ulceration) at the port site. Therefore, these infections were not considered to be CRBSI. The most common pathogens were *Escherichia coli* (n=16, 45.7%), *Klebsiella pneumoniae* (n=10, 28.6%), *Pseudomonas aeruginosa* (n=6, 17.1%), *Enterococcus* (n=3, 8.6%), and *Candida tropicalis* (n=3, 8.6%).

**Figure 1:**
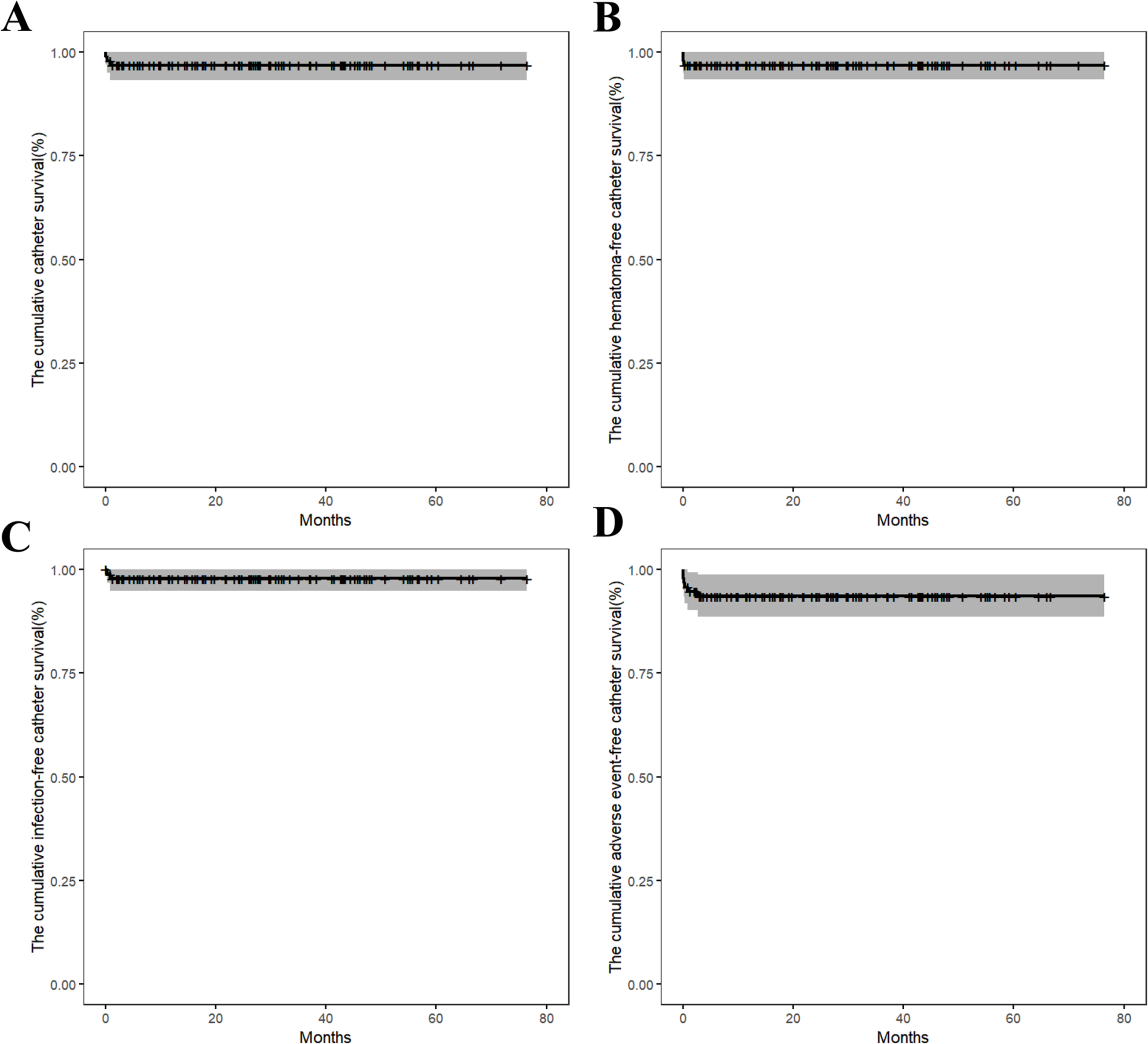
**(A)** The cumulative catheter survival; **(B)** The cumulative hematoma-free catheter survival; **(C)** The cumulative infection-free catheter survival; **(D)** The cumulative adverse event-free catheter survival rates (hematoma, infection, thrombosis and dysfunction).

In the first questionnaire one month after catheter insertion, 32 patients (34.0%) reported experiencing NRS (Numeric Rating Scale) grade 1–4 pain during TIVAP implantation. Three of 94 patients (3.2%) required premature TIVAPs removal due to localized bleeding complications and/or CRBSI. No patient requested TIVAP removal during chemotherapy, and no patient reported that the TIVAP interfered with daily activities (showering and bathing). The median survival of the TIVAP was 26.6 months (range: 0.1-76.4 months). The 2-year of cumulative catheter survival rate was 96.8% ± 1.82%. The 2-year of cumulative hematoma-free catheter survival rates was 96.8% ± 1.81%. The 2-year of cumulative infection-free catheter survival rates was 97.8% ± 1.51%. The 2-year of cumulative adverse event-free catheter survival rates (hematoma, infection, thrombosis and dysfunction) was 93.5% ± 2.55% (**Figure 1**).

## Discussion

Peripherally inserted central catheters (PICCs) have long been widely used in cancer patients, particularly those with acute leukemia, where they serve as essential vascular access for chemotherapy administration. First, a specially trained nursing team can place a PICC in the ward, which is available in many hospitals (5). Second, PICCs have minimal restrictions regarding platelet counts and coagulation parameters, allowing immediate implantation even in acute leukemia patients with thrombocytopenia and coagulopathy.

However, the overall incidence of adverse reactions with TIVAPs is lower than with PICCs (6), while providing comfortable, convenient long-term venous access for cancer patients (12, 13). Consequently, TIVAPs are increasingly used in acute leukemia patients. Previous randomized controlled trials (RCTs) demonstrated higher complication rates with PICCs versus TIVAPs in non-hematologic malignancies (6). However, the evidence regarding the safety and feasibility of TIVAP in patients with acute leukemia remains limited. In our study, the first-attempt TIVAP implantation success rate was 100%, comparable to first-attempt success rates (90%-99.3%) in prior adult studies (14, 15). This may be attributed to our surgeons’ interventional department background and extensive vascular puncture experience.

During follow-up, the overall complication rate after TIVAP implantation was 7.4% in our study. In a cumulative total of 82430 catheter days, there were 0.08 postoperative complications per 1000 catheter days. This outcome was similar to previous studies reporting 5.08% overall complication rates and 0.11 complications per 1000 catheter days (15, 16). However, in one RCT, the postoperative complication rate following TIVAP implantation in adults with non-hematologic malignancies was 13.1% (26/129) (6). The lower overall complication rate after TIVAP implantation in our cohort may be attributed to the use of chest wall ports. A meta-analysis suggested that chest wall ports may reduce overall risk of complications and risk of catheter-related thrombosis compared to arm ports (17). Complications included catheter-related adverse events (CR-DVT, exit-site infection, pocket infection, CRBSI, and occlusion) and mechanical complications (insertion failure and accidental removal).

In our study, the postoperative non-infectious complications, such as local subcutaneous hematoma was only 3.2%, similar to the 2.2% rate reported among 700 adult cancer patients (18). Other study had shown that the local subcutaneous hematoma was 2.5% for a pediatric non-hematologic malignancy cohort after TIVAP implantation, which was also similar to our group (19). However, both studies enrolled patients with non-malignant hematological diseases, and most maintained normal platelet levels pre-TIVAP implantation. Our cohort exclusively comprised acute leukemia patients with a median pre-implantation platelet count of 76 × 10^9^/L, and 24.5% had platelets counts below 50 × 10^9^/L. It is generally accepted that the platelet count should be over 50 × 10^9^/L prior to insertion of a catheter other than a PICC, and the INR <1.5 (20). However, it should not be overlooked that there are no published prospective, randomized studies to support or negate the theory that the level of platelet count at time of CVC insertion should be maintained above 50 × 10^9^/L to reduce potential for significant bleeding problems (21). Furthermore, a previous study evaluated the safety of totally implantable venous access port implantation under interventional radiology guidance in patients with severe thrombocytopenia (platelet count <50×10^9^/L). With preoperative or intraoperative platelet transfusion support, all patients, including those with platelet counts as low as <8×10^9^/L, successfully underwent the procedure, and no postoperative bleeding complications occurred (22). Thus, for patients with acute leukemia, preoperative ultrasound guidance is employed to identify optimal vasculature and minimize tissue trauma. During the procedure, meticulous compression is applied to achieve hemostasis, and bleeding is closely monitored. Postoperative platelet transfusion is utilized to further mitigate the risk of bleeding. When these preoperative, intraoperative, and postoperative measures are diligently implemented, TIVAP implantation is a feasible and safe procedure.

Infection is the most common complication after TIVAP implantation, which mostly occurs in the early stage of port insertion, and is also the main cause of early TIVAP removal (23–25). Multiple studies report that the rate of infection after TIVAP implantation in adult patients with non-hematologic malignancies is 2.2% to 9% (6, 26–30). Zhang et al. showed that the rate of overall infection was 4.0% in 223 hematologic malignancies patients after TIVAP implantation, which was comparable to the rate reported for patients with non-hematologic malignancies (31). Severe neutropenia (absolute neutrophil count <500 cells/μL) constitutes a significant risk factor for catheter-related infections in oncological patients (32–34). However, in our study, the incidence of CRBSI after TIVAP implantation in patients with acute leukemia was 2.1%. Our result suggested that the incidence of overall infection after TIVAP implantation was not higher than rates reported for both non-hematologic and hematologic malignancies (primarily lymphoma). This study supports the widely reported conclusion that TIVAPs maintain low infection rates, despite the frequent immunocompromised status of the patients requiring them. It was possible that the lower rate of overall infection after TIVAP implantation in our group may be explained by the lower proportion of patients with severe neutropenia (16.0%). Moreover, infection rates of TIVAPs are likely lower than those of external catheters, attributable to less frequent flushing, eliminated patient-administered maintenance, and reduced contamination risk during non-use periods (35). In addition, nearly 40% of patients in our cohort received antibiotic therapy for active infection prior to TIVAP implantation, which may have contributed to the low incidence of post-TIVAP implantation infection. Meticulous sterile technique during insertion and proper post-placement care remained the most effective infection prevention measures. However, when patients with TIVAPs develop infections, particularly CRBSI, despite antibiotic efficacy reaching 78-86%, TIVAP removal remains a critical intervention (36).

Based on previous literature, late complications occur in approximately 6% of cases, most commonly infection, followed by catheter dysfunction, CR-DVT and so on (37). CR-DVT represents a distinct clinical entity within venous thromboembolism, with multifactorial pathogenesis, and several risk factors have been suggested. Stagnant flow, vascular injury, stimulation of anti-tumor drugs and the blood hypercoagulability associated with both malignant disease and the catheter itself, are classic contributors to venous thrombosis (38). In our study, the incidence of CR-DVT was 1.1%, which was lowered to previous reports, ranging from 1.6% to 3.3% (39–44). Although research data on CR-DVT incidence in acute leukemia patients after TIVAP implantation were scarce, our CR-DVT incidence was comparable to or lower than rates in non-hematologic malignancies and non-acute leukemia hematologic malignancies. Our findings suggest that TIVAP implantation in acute leukemia patients does not appear to increase CR-DVT risk. However, when CR-DVT occurred, we implemented the protocol described by Baskin et al., enabling TIVAP retention without elevating adverse event incidence (45).

Catheter dysfunction, defined as inability to withdraw blood with or without impaired fluid infusion, may result from catheter thrombosis, fibrin sheath formation, or catheter tip adherence to the vascular wall (46). Previous studies have shown that the incidence of catheter dysfunction after TIVAP placement ranged from 0.5% to 13.2% (6, 15, 47). The incidence of catheter dysfunction after TIVAP implantation in different centers varies greatly, which may be related to differing definitions of catheter dysfunction and the heterogeneity of the study population. In our study, only one patient (1.1%) developed this complication, which might be related to intracavitary thrombotic obstruction that prevented blood suction and fluid injection. Although catheter functional complications after TIVAP implantation are not common, the occurrence of such complications may increase the risk of CR-DVT (48). Therefore, once the event of catheter dysfunction occurs, clinicians should address it promptly. Furthermore, no rare complications such as catheter or port membrane injury leading to catheter leakage, venous displacement, or catheter rupture were observed in our study.

TIVAP is safe and provides long-term benefits for patients with acute leukemia, with a low overall complication rate. In addition, TIVAP has less impact on certain activities of daily living, such as work, bathing, among others. TIVAP is a reasonable, beneficial, long-term, and safe intervention for patients with acute leukemia requiring repeated blood transfusions or frequent inpatient chemotherapy.

However, there are several limitations to this study. Firstly, this is a single-center, small-sample, retrospective analysis. Further prospective, multi-institutional, large-sample studies are needed to further elucidate the safety and feasibility of TIVAP in patients with acute leukemia. Moreover, this study does not compare the difference in complication rates between TIVAP and other modes of central access such as PICC. Next, we will further compare the application of TIVAP and PICC in acute leukemia. In addition, due to the small number of cases and complications, we could not analyze the relationship between clinical characteristics (such as neutrophil count and platelet levels) and these events.

## Conclusion

To our knowledge, this is one of the few studies exploring the feasibility and safety of TIVAP in patients with acute leukemia. Our research shows that TIVAP can provide a safe and effective system for long-term intravenous treatment of patients with acute leukemia. These data can be utilized by clinicians to help guide clinical decision making when considering TIVAP placement for their acute leukemia patients.

## Data Availability

The raw data supporting the conclusions of this article will be made available by the authors, without undue reservation.
